# Alterations in pain processing circuitries in episodic migraine

**DOI:** 10.1186/s10194-021-01381-w

**Published:** 2022-01-15

**Authors:** Tiffani J. Mungoven, Kasia K. Marciszewski, Vaughan G. Macefield, Paul M. Macey, Luke A. Henderson, Noemi Meylakh

**Affiliations:** 1 School of Medical Sciences (Neuroscience), Brain and Mind Centre, University of Sydney, Camperdown, NSW 2050 Australia; 2 Baker Heart and Diabetes Institute, Melbourne, VIC 3004 Australia; 3 UCLA School of Nursing and Brain Research Institute, University of California, Los Angeles, California 90095 USA

**Keywords:** Cortical pain modulation, Brainstem pain modulation, Functional connectivity, PPI, Migraine, Orofacial pain, Dorsolateral prefrontal cortex, Hypothalamus, Spinal trigeminal nucleus

## Abstract

**Background:**

The precise underlying mechanisms of migraine remain unknown. Although we have previously shown acute orofacial pain evoked changes within the brainstem of individuals with migraine, we do not know if these brainstem alterations are driven by changes in higher cortical regions. The aim of this investigation is to extend our previous investigation to determine if higher brain centers display altered activation patterns and connectivity in migraineurs during acute orofacial noxious stimuli.

**Methods:**

Functional magnetic resonance imaging was performed in 29 healthy controls and 25 migraineurs during the interictal and immediately (within 24-h) prior to migraine phases. We assessed activation of higher cortical areas during noxious orofacial heat stimulation using a thermode device and assessed whole scan and pain-related changes in connectivity.

**Results:**

Despite similar overall pain intensity ratings between all three groups, migraineurs in the group immediately prior to migraine displayed greater activation of the ipsilateral nucleus accumbens, the contralateral ventrolateral prefrontal cortex and two clusters in the dorsolateral prefrontal cortex (dlPFC). Reduced whole scan dlPFC [Z + 44] connectivity with cortical/subcortical and brainstem regions involved in pain modulation such as the putamen and primary motor cortex was demonstrated in migraineurs. Pain-related changes in connectivity of the dlPFC and the hypothalamus immediately prior to migraine was also found to be reduced with brainstem pain modulatory areas such as the rostral ventromedial medulla and dorsolateral pons.

**Conclusions:**

These data reveal that the modulation of brainstem pain modulatory areas by higher cortical regions may be aberrant during pain and these alterations in this descending pain modulatory pathway manifests exclusively prior to the development of a migraine attack.

## Background

Migraine is a common debilitating neurological disorder, characterized by severe attacks of pulsating head pain with the accompaniment of symptoms such as photophobia, phonophobia, nausea and vomiting. Although the precise underlying mechanisms remain poorly understood, there is growing evidence that changes within the brain itself may be critical for the initiation of a migraine attack [1–3]. One emerging hypothesis is that brainstem sensitivity oscillates across the migraine cycle, regulating the brainstem region that receives orofacial noxious afferents: the spinal trigeminal nucleus (SpV). More specifically, altered modulation of the SpV by descending circuits can initiate a migraine by either increasing on-going neural traffic within the SpV or by allowing an external trigger to increase SpV activity; both of which would increase activation of cortical areas and elicit head pain [4].

Consistent with this brainstem oscillation hypothesis, we recently reported that in episodic migraineurs, acute noxious orofacial stimulation evoked greater activation of the SpV compared with controls during the 24-h period immediately prior to a migraine attack and not during the interictal period [5]. This increased activation occurred despite the overall perceived pain intensities being no different to that of the control group [5]. In addition, we found that resting state functional connectivity strengths between the SpV and rostral ventromedial medulla (RVM) were reduced only during this same period [5]. The most well-described brainstem pain modulatory pathway involves the midbrain periaqueductal gray matter (PAG) - RVM - SpV circuit [6–11] and our results suggest that migraine is associated with fluctuations in descending pain modulatory pathways over the migraine cycle [5].

Brainstem pain modulatory regions are themselves modulated by higher brain regions, as observed by experimental animal investigations, which have revealed that PAG sensitivity is regulated by the hypothalamus and that hypothalamus sensitivity itself is regulated by the cerebral cortex [12–14]. One emerging line of evidence is that the hypothalamus is critical for migraine generation [15, 16] and we have previously shown that immediately prior to a migraine, the lateral hypothalamus displayed decreases in resting regional cerebral blood flow and altered connectivity with the PAG, dorsomedial pons, SpV and RVM [17]. Whilst we have shown acute orofacial pain evoked changes within the brainstem of individuals with migraine, we do not know if these brainstem alterations are driven by changes in higher brain centers such as the hypothalamus and/or areas of the prefrontal cortex (PFC). It might be that higher cortical and hypothalamic areas may contribute to the initiation and maintenance of migraine pain through their modulation of descending pain modulatory pathways.

The aim of this study is to extend our previous investigation [5] to determine if higher brain centers display altered activation patterns in migraineurs during acute orofacial noxious stimuli. We hypothesize that migraineurs will display altered activation patterns in response to acute orofacial noxious stimuli in cortical pain modulatory regions such as the PFC and hypothalamus. Furthermore, we aim to determine if any activation differences are associated with altered connectivity with brainstem pain modulatory regions, namely the PAG. We also hypothesize that migraineurs will show altered functional connectivity between higher cortical brain regions involved in pain modulation and the PAG, in particular during the 24-h period immediately prior to a migraine attack.

## Methods

### Subjects

Twenty-five subjects with episodic migraine (6 males, mean ± SEM age: 29.6 ± 2.0 years, range 19–54) and 29 pain-free controls (10 males, mean ± SEM age: 26.4 ± 1.4 years, range 19–57) were recruited for the study from the general population using an advertisement. Migraine subjects were diagnosed according to the criteria set by the International Classification of Headache Disorders (ICHD), 3rd edition, sections 1.1 and 1.2 [18]. Four migraineurs reported experiencing aura with their migraines, and the remaining 21 reported no aura. Of the 25 migraineurs, 20 were placed into an *interictal* group as they were scanned during the interictal period, i.e. at least 72 h after and 24 h prior to a migraine attack. Of the 25 migraineurs, 7 migraineurs were placed into an *immediately prior to migraine* group since they were scanned during the 24-h period immediately before a migraine. Two migraineurs were scanned during both an interictal and immediately prior to a migraine phase. There were no significant differences in age (*t-*test; *p* > 0.05), or gender composition (X^2^ test, *p* > 0.05) between the three groups.

**Fig. 1:**
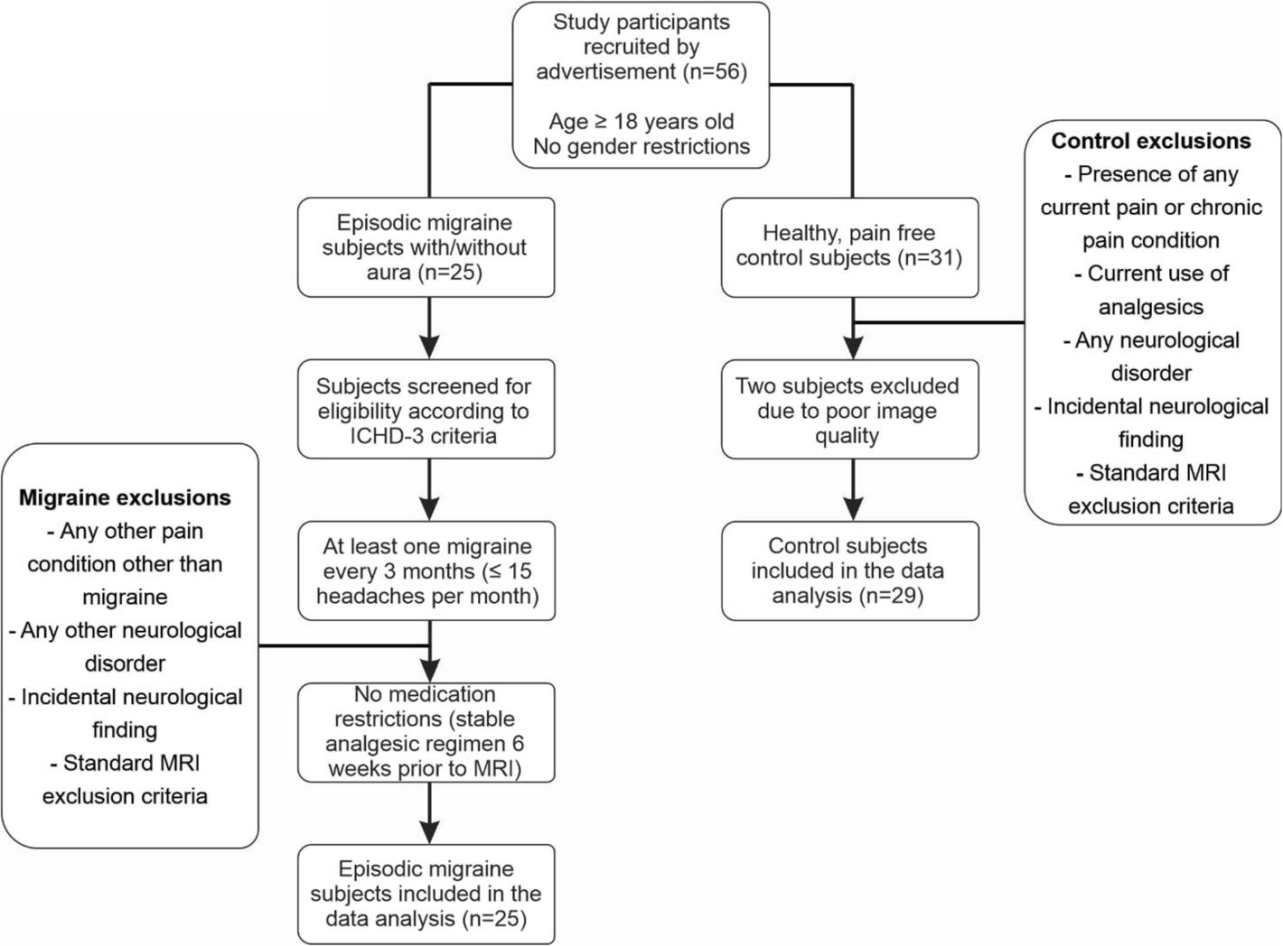
Flow chart of the inclusion and exclusion criteria of control and episodic migraine study participants

All migraine subjects indicated the pain intensity (6-point visual analog scale; 0 = no pain, 5 = most intense imaginable pain) and drew the facial distribution of pain they commonly experienced during a migraine attack. Additionally, each subject described the qualities of their migraines and indicated any current treatments used to prevent or abort a migraine once initiated. Exclusion criteria for controls were the presence of any current pain or chronic pain condition, current use of analgesics, and any neurological disorder. Exclusion criteria for migraineurs were any other pain condition other than migraine or any other neurological disorder. No migraineur was excluded based on their medication use and no migraine or control subject had an incidental neurological finding that resulted in their exclusion from the study (Fig. 1). Informed written consent was obtained for all procedures according to the Declaration of Helsinki seventh revision and local Institutional Human Research Ethics Committees approved the study. Data from several subjects used in this investigation have been used in previous investigations [5, 17, 19–21].

### MRI acquisition

In all control subjects, before entering the magnetic resonance imaging (MRI) scanner, a 3 × 3 cm MRI-compatible thermode (Medoc, Ramat Yishai, Israel) was placed on the right side of the corner of the mouth covering the upper and lower lips. In migraineurs, the thermode was also placed on the right corner of the mouth except for 4 migraineurs in which it was placed on the left side, since they were the only migraineurs that most commonly experienced headaches on the left side. A thermode device was used to deliver nociceptive heat stimuli as it is a well-established, non-invasive experimental pain method with highly controlled temperatures and pain duration [22, 23]. A temperature that evoked moderate pain ratings was determined for each individual subject with a Thermal Sensory Analyzer (TSA-II, Medoc), from a resting temperature of 32 °C to temperatures at 0.5 °C intervals between 44 °C and 49 °C. Temperatures were randomly applied in 15 s intervals for 10 s during which each subject rated the pain intensity using a 10 point Computerised Visual Analog Scale (CoVAS, Medoc; 0 = no pain, 10 = worst imaginable pain). The temperature at which individuals indicated a pain intensity rating of approximately 6 out of 10, was used for the remainder of the experiment.

**Fig. 2:**
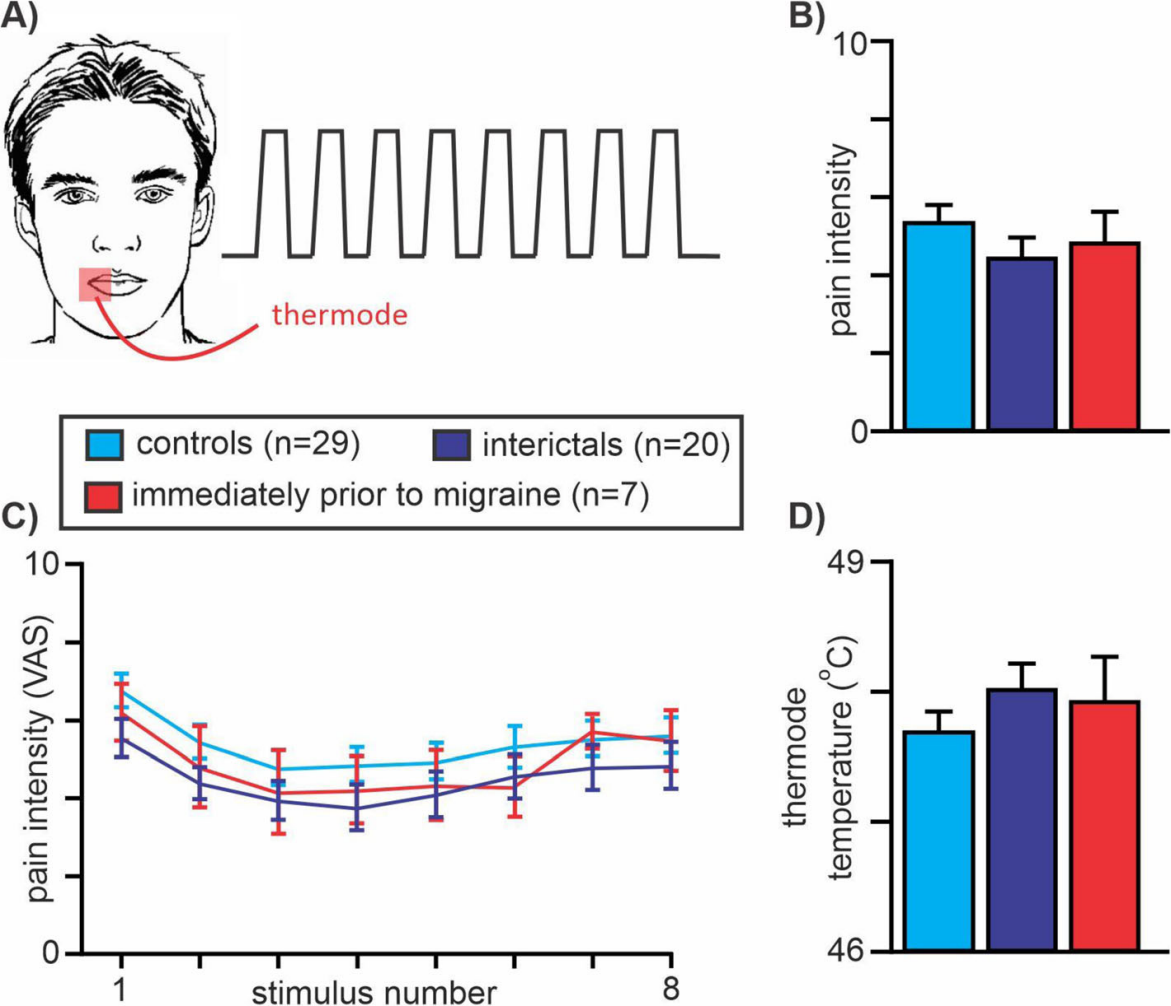
**A** Eight acute noxious thermal stimuli were delivered to the corner of the mouth in controls, migraineurs during the interictal phase and in migraineurs in the 24 h immediately prior to a migraine headache; (**B)** Mean ± SEM pain intensity ratings over the 8 noxious stimuli for each group; **(C)** Mean ± SEM pain intensity ratings for each of the 8 noxious stimuli in each group; **(D)** Mean ± SEM administered thermode temperatures for each of the three groups. Note there were no significant differences in pain intensity rating or thermode temperatures between any of the groups

All subjects then lay supine on the bed of a 3 T MRI scanner (Philips, Achieva) with their head immobilized in a 32-channel head coil. With each subject relaxed and at rest, a high-resolution 3D T1-weighted anatomical image set covering the entire brain was collected (turbo field echo; field of view 250 × 250 mm, raw voxel size 0.87mm^3^, repetition time 5600 ms, echo time 2.5 ms, flip angle 8^o^). Following this, a series of 140 gradient-echo echo planar functional MRI image volumes with blood oxygen level-dependent (BOLD) contrast was collected with each image volume covering the entire brain (38 axial slices, repetition time 2500 ms, raw voxel size 1.5 × 1.5 × 4.0 mm thick). During this functional magnetic resonance imaging (fMRI) scan, following a 30-volume baseline period, 8 noxious thermal stimuli were delivered (Fig. 2A). Each noxious stimulus was delivered for 15 s (including ramp up and down periods of 2.5 s each), followed by a 15 s baseline (32 °C) period. During each period of noxious stimulation, the subject was asked to rate the pain intensity online using the CoVAS.

### MRI image preprocessing

In the 4 migraineurs in whom the thermode was placed on the left side of the mouth (2 were scanned during the interictal phase, 2 were scanned during both interictal and immediately prior to migraine phases), their MRI images were reflected in the X plane so that in all subjects the right side was ipsilateral to the delivered noxious thermal stimulus. Using SPM12 [24] and custom software, fMRI images were slice-timing corrected, motion corrected and the effect of motion on signal intensity was modelled and removed using LMRP detrending. Physiological (i.e. cardiovascular and respiratory) noise was then modelled and removed using the DRIFTER toolbox [25] and the images were then linear detrended to remove global signal intensity drifts. Each subject’s fMRI image set was co-registered to their own T1-weighted anatomical image. The T1 images were then spatially normalized to the Montreal Neurological Institute (MNI) template and the normalization parameters were applied to the fMRI images to place them in MNI space. Finally, the wholebrain images were smoothed using a 6 mm full-width half maximum (FWHM) Gaussian filter. In addition, prior to spatial normalization, using brainstem-specific isolation software (SUIT toolbox in SPM12) [26], a mask of the brainstem was created for each subject for both the T1 and fMRI image sets. Using these masks, the brainstem of the T1 and fMRI image sets were isolated and then spatially normalized to the SUIT brainstem template in MNI space. These brainstem-only image sets were then spatially smoothed using a 3 mm FWHM Gaussian filter. A small smoothing kernel was used to maintain spatial accuracy in small brainstem sites. Two control subjects were excluded from the final analysis due to poor image quality following preprocessing.

### Acute pain related signal intensity change analysis

Changes in signal intensity during the 8 noxious stimuli were determined using a repeated box-car model convolved with a canonical haemodynamic response function. Since we have already investigated changes within the brainstem [5], we will assess only those changes above the brainstem using the wholebrain images (cortical/subcortical images). Significant differences between the control group and those migraineurs scanned during the interictal phase (*n* = 20) were determined using a two-group random effects analysis in SPM12 (*p* < 0.05, false discovery rate [FDR] [27] corrected for multiple comparisons, minimum cluster size of 10 contiguous voxels, age and gender as nuisance variables). In addition, significant differences between controls and migraineurs scanned during the 24-h period immediately prior to a migraine headache (*n* = 7) were also determined using a two-group random effects analysis (*p* < 0.05, FDR corrected, minimum cluster size 10, age and gender as nuisance variables). Significant clusters were overlaid onto a mean T1 anatomical and beta values for significant clusters were extracted and the mean ± SEM plotted for all three groups (controls, migraine interictal, migraine immediately prior to migraine). If a significant cluster was derived from the control versus interictal analysis, then mean beta values were compared between control and immediately prior to migraine groups for that cluster using two-sample *t*-tests (*p* < 0.05, Bonferroni corrected for multiple comparisons). In this instance, differences between controls and interictals were not compared to avoid statistical double-dipping.

### Cortical/subcortical whole scan connectivity change analysis

To assess the potential descending influences onto brainstem pain modulatory circuits, we used four of the clusters that displayed significant differences in the initial acute pain activation analysis (ipsilateral nucleus accumbens [NAc], contralateral ventrolateral prefrontal cortex [vlPFC] and two clusters in the contralateral dorsolateral prefrontal cortex [dlPFC]) and performed two different connectivity-based analyses. *Whole scan connectivity:* for each of these clusters we firstly performed a whole scan functional connectivity analysis. That is, we extracted the mean signal intensity change from the cortical/subcortical image sets for each of the four clusters. Instead of using a regressor based model, we used the signal intensity changes extracted from a particular seed as the regressor. This regressor was not convolved with a haemodynamic response function. We then performed a voxel-by-voxel connectivity analysis over the entire fMRI scan, creating four brain maps (one map for each seed region) with each voxel value indicating the connectivity strength for each subject. Significant differences in whole scan connectivity strengths between controls and interictals and between controls and immediately prior to migraine were determined using two-group random effects analyses (*p* < 0.05, FDR corrected, minimum cluster size 10 voxels, age and gender nuisance variables).

### Cortical/subcortical pain-related connectivity change analysis

In addition to whole scan connectivity changes, we assessed pain-related changes in connectivity strengths for each of the four clusters. We used a psychophysiological interaction (PPI) analysis technique in SPM12 which allows for examination of the interaction between the signal covariations of a physiological variable (four seeds) and a psychological variable (noxious orofacial stimulation) [28, 29]. The resultant brain maps provide an indicator of the degree and direction to which connectivity changes during the noxious stimulus periods compared with the baseline periods. Significant differences in pain-related changes in connectivity strengths between controls and interictals and between controls and immediately prior to migraine were determined using two-group random effects analyses (*p* < 0.05, FDR corrected, minimum cluster size 10 voxels, age and gender nuisance variables).

### Dorsolateral PFC and hypothalamus brainstem specific connectivity change analysis

The cortical/subcortical whole scan and pain-related changes in connectivity analysis revealed that only one of the four clusters, the dlPFC [at Z level + 44], showed significant differences between controls and migraineurs. Since this brain region has been heavily implicated in pain modulation [30, 31], we focussed our subsequent analysis on this dlPFC region. Furthermore, it is thought that the dlPFC modulates the brainstem directly or via projections to the hypothalamus [31] and our whole scan connectivity analysis revealed a significant change in dlPFC connectivity with the hypothalamus in migraineurs. Given this we determined whether changes in whole scan and pain-related changes in connectivity strengths between the dlPFC and brainstem and between the hypothalamus and brainstem were altered in migraineurs. Using the brainstem specific fMRI images, we used the dlPFC and hypothalamic seeds to assess whole scan and pain-related changes in connectivity in the same analysis procedures described above.

Differences in whole scan and pain-related changes in brainstem connectivity between the migraine groups and controls were determined using two-group random effects analyses (*p* < 0.001, uncorrected, minimum cluster size 10 voxels, age and gender nuisance variables). To reduce the likelihood for Type 1 errors we performed cluster level correction for multiple comparisons. Significant clusters were overlaid onto a standard brainstem template and mean ± SEM connectivity strengths plotted for each cluster for each group. If a significant cluster was derived from the control versus interictal analysis, then connectivity strength values were compared between control and immediately prior to migraine groups for that cluster using two-sample *t*-tests (*p* < 0.05). The location of brainstem clusters was identified using the Atlas of the Human Brainstem [32] and the Duvernoy Brainstem Atlas [33].

## Results

### Migraine characteristics

Using a self-report questionnaire following an episodic migraine diagnosis screening, of the 25 migraineurs, 11 reported that their headaches occurred most commonly on the right side, while 4 reported more on the left and the remaining 10 reported that they would occur on either side (see Table 1 for migraineur characteristics). Migraine subjects most frequently described their migraine pain as “throbbing,” “sharp,” and/or “pulsating” in nature and indicated that “stress,” “lack of sleep,” and/or “bright light” most often triggered their migraine attacks. The mean estimated frequency of migraine attacks was 16.4 ± 1.9 per year, mean length of time since the onset of migraine attacks (years suffering) 15.4 ± 2.3 years, and mean pain intensity of migraines 3.7 ± 0.2 on a 6-point visual analog scale. Although 16 of the 25 migraineurs were taking some form of daily medication (mostly the oral contraceptive pill; 10 migraineurs), none of the migraine subjects were taking prophylactic medication prescribed for migraine.

**Table 1:** Migraine subject characteristics. M: male; F: female; B: bilateral; L: left; R: right; OCP: oral contraceptive pill

*Subject*	*Age*	*Sex*	*Years suffering*	*Pain side*	*Aura*	*Frequency (per month)*	*Intensity (0–5)*	*Medication taken during migraine*	*Daily medication*
1	31	F	25	R	Y	> 3	3–4	paracetamol	–
2	53	M	15	B	N	> 3	4	ibuprofen, paracetamol	–
3	24	F	20	B	N	> 3	4	ibuprofen, paracetamol	OCP, budesonide/formoterol
4	26	F	12	R	N	2	3–4	ibuprofen	OCP
5	27	F	12	R	Y	1	4	ibuprofen	OCP
6	23	F	4	R	N	> 3	4	triptan	OCP, metformin hydrochloride
7	25	F	12	L	N	> 3	3	aspirin, rizatriptan	desvenlafaxine
8	21	F	1.5	L	N	> 3	3	ibuprofen, paracetamol, codeine	OCP
9	26	F	1	B	N	> 3	5	paracetamol	OCP
10	29	F	13	R	N	1	2.5	ibuprofen	zopiclone
11	26	F	5	R	N	1	2	aspirin, codeine, ibuprofen	OCP
12	23	F	6	R	N	1	3–4	ibuprofen	OCP
13	23	F	10	B	N	0.5–1	4	ibuprofen, codeine	OCP
14	46	F	15–20	B	N	1	3	sumatriptan	–
15	41	F	40	B	N	2	4	sumatriptan	–
16	26	M	15	B	N	> 3	2	TCE, paracetamol, codeine	–
17	23	M	3–4	B	N	0.5–1	3.5	paracetamol, codeine	–
18	23	M	4–5	B	N	0.5–1	4	paracetamol	–
19	55	F	40	R	N	0.5–1	3–4	sumatriptan	telmisartan
20	26	M	20	R	N	0.5–1	4	metamizole	carbamazepine
21	49	F	30	B	N	0.5–1	5	rizatriptan, paracetamol	–
22	27	M	4	B	N	0.5–1	4	ibuprofen	SSRI
23	28	F	25	R	Y	0.25	5	ibuprofen, codeine	methylphenidate
24	24	F	13	R	Y	> 3	5	TCL, paracetamol, codeine	–
25	19	F	4–5	B	N	> 3	3	–	Lexapro, OCP

### Pain ratings

The overall pain intensity ratings during the 8 brief noxious heat stimuli were similar in all 3 groups (mean ± SEM VAS: controls 5.4 ± 0.4; interictal 4.5 ± 0.5; immediately prior to migraine 4.9 ± 0.7; two-tailed *t*-test, all *p* > 0.05). In addition, there was also no significant difference in the applied thermode temperature used to evoke these pain levels between groups (mean temperature: controls 47.7 ± 0.2 °C; interictal 48 ± 0.2 °C; immediately prior to migraine 47.9 ± 0.3 °C; Fig. 2).

### Acute pain related signal intensity changes

**Fig. 3:**
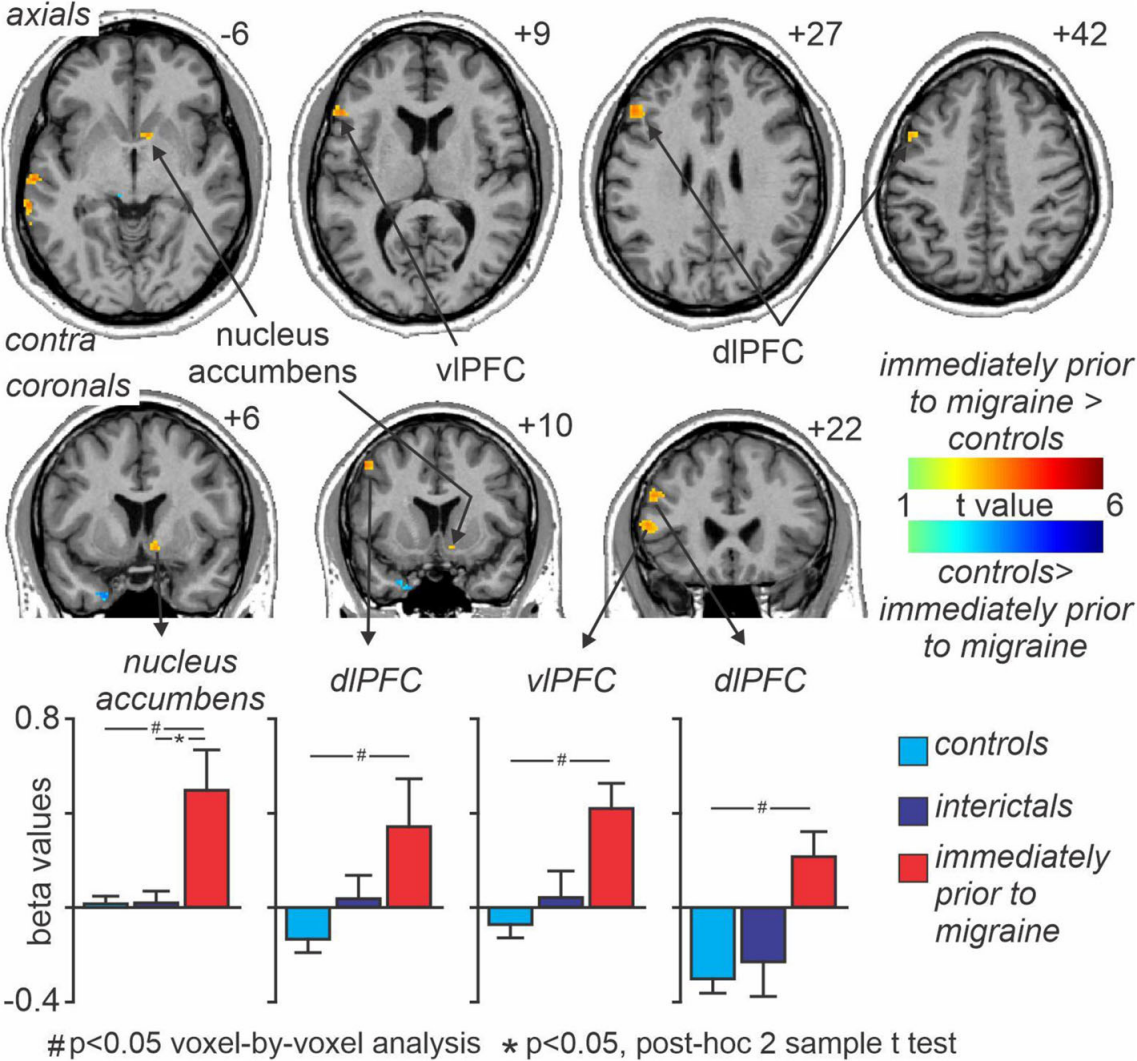
Significant differences in signal intensity changes during 8 noxious thermal stimuli in migraineurs immediately prior to a migraine headache compared with controls. Significant clusters are overlaid onto a mean T1-weighted anatomical image. Slice locations in Montreal Neurological Institute space are indicated to the top right of each slice. Note that signal increase changes were significantly greater in four main regions; the ipsilateral nucleus accumbens, contralateral ventrolateral prefrontal cortex (vlPFC) and two clusters in the dorsolateral prefrontal cortex (dlPFC). Plots of mean (±SEM) beta values (effect sizes) for each of these four clusters revealed that acute orofacial pain evoked significant signal intensity increases in migraineurs only during the 24-h period immediately prior to migraine, that is signal changes were not different between controls and migraineurs during the interictal phase

Across all subjects, acute noxious stimuli evoked significant signal intensity increases in a number of brain regions, including the insula, cingulate cortex, primary and secondary somatosensory cortices and decreases in the medial prefrontal and posterior parietal cortices and in the precuneus. Analysis of acute pain evoked changes in activation between groups revealed no significant differences between controls and migraineurs during the interictal phase. However, comparison of migraineurs immediately prior to a migraine revealed significant increases in a number of brain regions including the ipsilateral NAc, the contralateral vlPFC and two clusters in the dlPFC as well as the posterior parietal cortex and temporal cortex (Fig. 3, Table 2). Extraction of the magnitude of signal changes (beta values) also revealed that changes in signal within the four clusters: ipsilateral NAc, the contralateral vlPFC and two clusters in the dlPFC, did not change during the interictal period compared with controls (mean ± SEM beta values in controls, interictal, immediately prior to migraine: NAc 0.01 ± 0.04, 0.01 ± 0.07, *p* = 0.99, 0.51 ± 0.17, *p* < 0.001; vlPFC -0.06 ± 0.06, 0.04 ± 0.12, *p* = 0.43, 0.43 ± 11, *p* = 0.001; dlPFC [Z level + 28] -0.13 ± 0.06, 0.04 ± 0.11, *p* = 0.14, 0.35 ± 0.08, *p* < 0.001; dlPFC [Z level + 44] -0.29 ± 0.07, − 0.22 ± 0.15, *p* = 0.64, 0.24 ± 0.09, *p* = 0.001; all control versus interictal *p* > 0.05).

**Table 2:** Montreal Neurological Institute (MNI) coordinates, cluster size and t-score for regions with greater signal intensity changes

Brain region	MNI coordinate			cluster size	t-score
	x	y	z		
ventrolateral prefrontal cortex	−46	40	−4	16	3.92
	−52	24	10	29	3.76
posterior parietal cortex	−66	−48	−2	75	5.27
temporal cortex	−62	−20	−6	22	3.91
dorsolateral prefrontal cortex	−48	24	28	55	3.88
	−48	10	44	20	3.77
nucleus accumbens	10	6	−10	23	3.53

### Cortical/subcortical whole scan connectivity changes

**Fig. 4:**
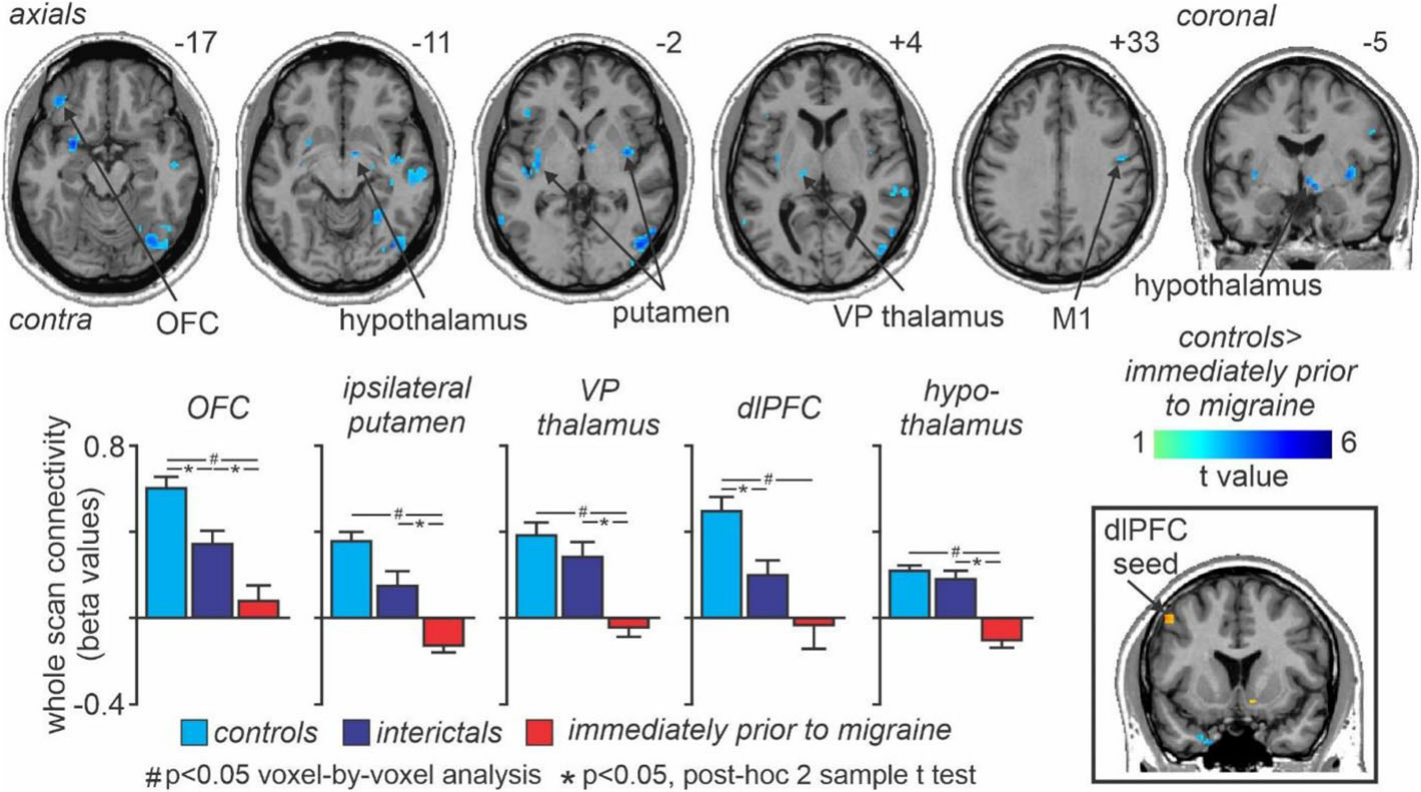
Whole scan connectivity: Significant differences in contralateral dorsolateral prefrontal cortex (dlPFC) whole scan connectivity between controls and migraineurs in the period immediately prior to a migraine headache. Significant clusters are overlaid onto a mean T1-weighted anatomical image. Slice locations in Montreal Neurological Institute space are indicated to the top right of each slice. The dlPFC seed is shown in the lower right inset. Note that connectivity strengths were significantly reduced in a number of brain regions including the orbitofrontal cortex (OFC), putamen, ventroposterior (VP) thalamus, primary motor cortex (M1) and the hypothalamus. Plots of mean (±SEM) beta values (effect sizes) revealed that whole scan connectivity values decreased significantly in migraineurs only during the 24-h period immediately prior to migraine, that is, they were not different between controls and migraineurs during the interictal phase. Beta values indicate the strength of functional connectivity between the dlPFC and respective brain regions

Whole scan connectivity analysis revealed no significant differences between the control and interictal groups for any of the four clusters, i.e. NAc, vlPFC, dlPFC [Z + 28], dlPFC [Z + 44]. Similarly, comparison of whole scan connectivity between control and immediately prior to migraine groups revealed no significant differences for the NAc, vlPFC or dlPFC [Z + 28] clusters, however significant differences did occur in a number of brain regions for the dlPFC [Z + 44] cluster. Significantly reduced whole scan dlPFC connectivity strengths occurred in the contralateral orbitofrontal cortex (OFC), putamen, ventroposterior (VP) thalamus, hippocampus, dlPFC and the ipsilateral putamen, hypothalamus, primary motor cortex (M1) and posterior parietal cortex (Fig. 4, Table 3). Extraction of the magnitude of connectivity strength also revealed that changes in whole scan dlPFC connectivity within these clusters decreased significantly during the interictal phase in migraineurs compared with controls in the contralateral putamen (mean ± SEM connectivity strength values in controls, interictals, immediately prior to migraine: 0.13 ± 0.01, 0.05 ± 0.02, *p* = 0.005, − 0.05 ± 0.01, *p* < 0.001), contralateral dlPFC (0.19 ± 0.02, 0.07 ± 0.03, *p* = 0.002, − 0.01 ± 0.04, *p* < 0.001), contralateral OFC (0.22 ± 0.02, 0.12 ± 0.02, *p* = 0.001, 0.03 ± 0.03, *p* < 0.001), and ipsilateral M1 (0.17 ± 0.02, 0.07 ± 0.03, *p* = 0.005, − 0.03 ± 0.05, *p* < 0.001), but did not significantly change during the interictal period in the contralateral VP thalamus (0.14 ± 0.02, 0.10 ± 0.02, *p* = 0.24, − 0.02 ± 0.02, *p* = 0.001), ipsilateral putamen (0.09 ± 0.02, 0.07 ± 0.02, *p* = 0.35, − 0.09 ± 0.02, *p* < 0.001), or the ipsilateral hypothalamus (0.08 ± 0.01, 0.06 ± 0.02, *p* = 0.39, − 0.04 ± 0.01, *p* < 0.001). Furthermore, in the contralateral VP thalamus, ipsilateral putamen, hypothalamus and OFC, whole scan connectivity strength values were significantly decreased immediately prior to migraine compared with the interictal phase in migraineurs. In no region was whole scan connectivity strengths increased in migraineurs compared with controls.

**Table 3:** Montreal Neurological Institute (MNI) coordinates, cluster size and t-score for regions with reduced whole scan and pain-related connectivity changes

Brain region	MNI coordinate			Cluster size	t-score
	x	y	z		
***Dorsolateral prefrontal cortex whole scan cortical/subcortical connectivity***					
putamen					
	−26	2	−14	50	5.63
posterior parietal cortex	−34	−14	−6	92	4.64
	36	−4	−2	71	4.80
hippocampus	36	−78	−12	340	5.57
hypothalamus	−62	−54	−4	37	4.59
orbitofrontal cortex	30	−18	−12	45	4.83
	6	−4	−8	27	4.80
ventral midbrain	−40	26	−4	63	4.74
primary motor cortex	−36	36	−16	32	4.55
dorsolateral prefrontal cortex	−6	−24	−10	34	4.35
	48	−6	32	21	4.22
	−36	14	28	22	4.05
***Dorsolateral prefrontal cortex whole scan brainstem connectivity***					
rostral ventromedial medulla	−1	−40	−51	12	3.54
***Dorsolateral prefrontal cortex pain-related changes in brainstem connectivity***					
subnucleus reticularis dorsalis/ spinal trigeminal nucleus/rostral ventromedial medulla	−3	−44	−54	141	5.86
spinal trigeminal nucleus	9	−42	−48	35	4.24
dorsomedial pons	13	−36	−38	101	5.34
dorsolateral pons	12	−31	− 31	66	4.30
***Lateral hypothalamus pain-related changes in brainstem connectivity***					
midbrain periaqueductal gray matter	−1	−35	−7	45	3.58
dorsolateral pons	7	−38	− 32	45	4.41
	−9	−40	−30	87	3.91
rostral ventromedial medulla	−3	−28	−41	10	4.00

### Cortical/subcortical pain-related connectivity changes

Analysis of noxious stimulus pain-related changes in connectivity strengths (PPI analysis) revealed no significant differences between the control and interictal groups or between the control and immediately prior to migraine groups for any of the four clusters. All control versus immediately prior to migraine *p* > 0.05 and all control versus interictal *p* > 0.05.

### Dorsolateral PFC and hypothalamus brainstem specific connectivity changes

Given that it is known that the dlPFC can modulate pain by either direct descending projections to the brainstem or indirectly via the hypothalamus, we determined whether there were any whole scan or pain-related changes in noxious-stimulus connectivity between the dlPFC [Z + 44] and the brainstem as well as between the hypothalamus (cluster derived from whole scan dlPFC analysis) and the brainstem. Comparison of control with immediately prior to migraine groups revealed no significant whole scan connectivity differences between either the dlPFC [Z + 44] or hypothalamus. In addition, whilst there were also no significant differences in brainstem whole scan connectivity between the hypothalamus and brainstem during the interictal phase, the dlPFC [Z + 44] displayed significantly reduced whole scan connectivity with a discrete region of the rostral ventromedial medulla (mean ± SEM connectivity strength values in controls, interictals, immediately prior to migraine: 0.06 ± 0.02, − 0.10 ± 0.05, *p* < 0.001, − 0.02 ± 0.03, *p* = 0.11) (Table 3).

**Fig. 5:**
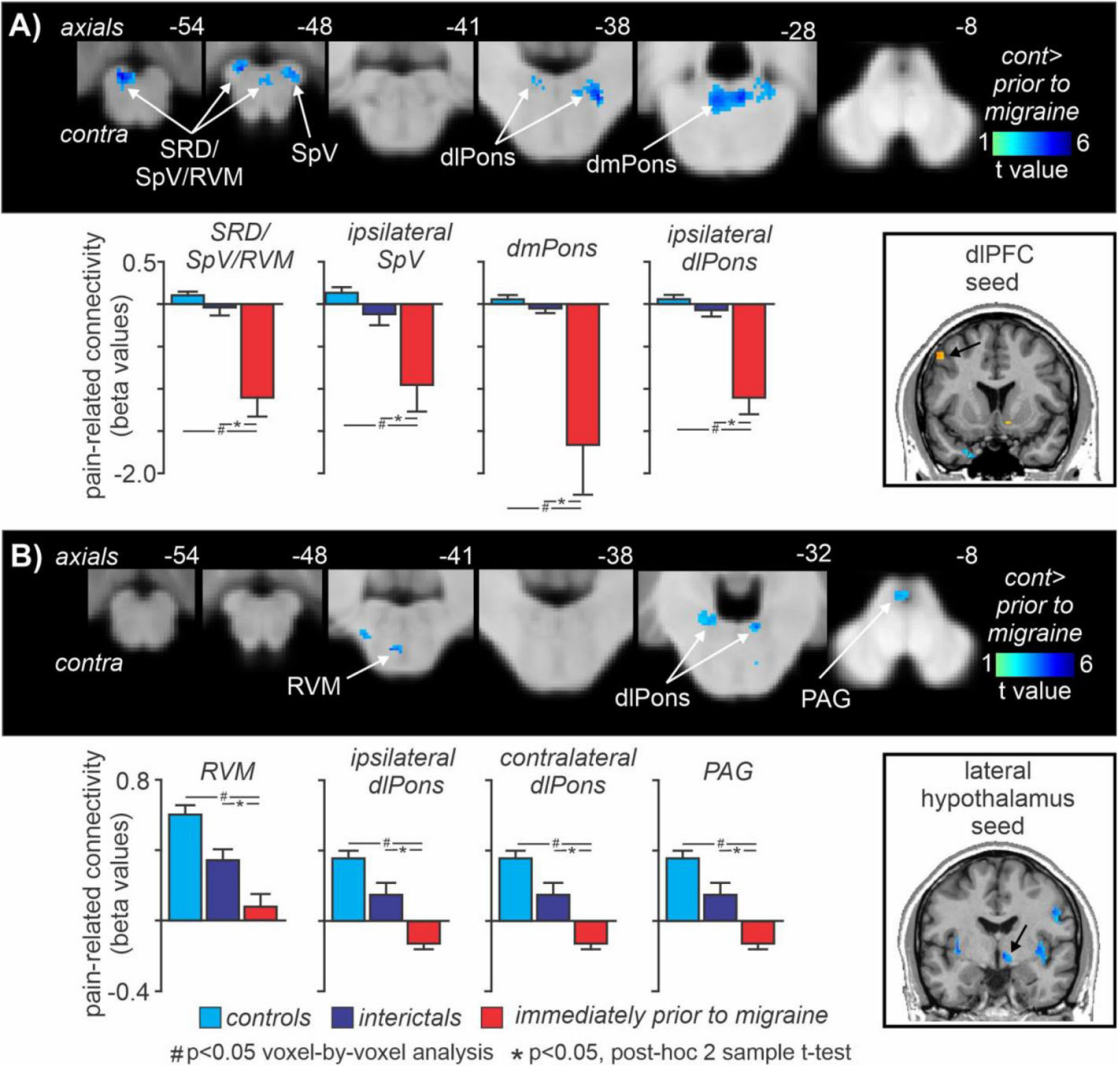
Pain-related connectivity: Significant differences in (**A)** contralateral dorsolateral prefrontal cortex (dlPFC) and (**B)** ipsilateral hypothalamus acute pain-evoked changes in connectivity (psychophysiological interaction analysis) between controls and migraineurs in the period immediately prior to a migraine headache. Significant clusters are overlaid onto a mean T1-weighted brainstem template image. Slice locations in Montreal Neurological Institute space are indicated to the top right of each slice. The dlPFC and hypothalamic seeds are shown in the lower right inset. Note that dlPFC pain-related connectivity strengths were significantly reduced in a number of brainstem regions including the dorsomedial pons (dmPons), dorsolateral pons (dlPons), spinal trigeminal nucleus (SpV), and a cluster encompassing the nucleus reticularis dorsalis (SRD)/SpV and rostral ventromedial medulla (RVM). More restricted pain-related hypothalamic connectivity changes occurred in the dlPons, RVM and also in the region of the midbrain periaqueductal gray matter (PAG). Plots of mean (±SEM) beta values (effect sizes) revealed that pain-related changes in connectivity decreased significantly in migraineurs only during the 24-h period immediately prior to migraine, that is they were not different between controls and migraineurs during the interictal phase

In striking contrast, whilst comparison of controls and interictal migraine groups revealed no significant differences in brainstem pain-related changes in connectivity for either the dlPFC or hypothalamus, comparison of controls with immediately prior to migraine group, revealed significant pain-related changes in connectivity differences within multiple brainstem sites. Significantly reduced dlPFC [Z + 44] changes in pain-related connectivity strengths occurred in the regions of the contralateral dorsolateral pons (dlPons), the dorsomedial pons (dmPons) spreading into the ipsilateral dlPons, the ipsilateral SpV and a larger cluster centered in the region of the subnucleus reticularis dorsalis (SRD) and extending to encompass the contralateral SpV and rostral ventromedial medulla (RVM) (Fig. 5A, Table 3). Extraction of the magnitude of pain-related connectivity strength changes also revealed that these changes were restricted to the period immediately prior to migraine and did not change during the interictal phase relative to controls (mean ± SEM PPI in controls, interictals, immediately prior to migraine: ipsilateral dlPons 0.05 ± 0.04, − 0.08 ± 0.10, *p* = 0.22, − 1.12 ± 0.19, *p* < 0.001; dmPons 0.05 ± 0.04, − 0.05 ± 0.08, *p* = 0.23, − 1.69 ± 0.58, *p* < 0.001; ipsilateral SpV 0.13 ± 0.04, − 0.14 ± 0.12, − 0.97 ± 0.31, *p* < 0.001; SRD/SpV/RVM 0.10 ± 0.03, − 0.06 ± 0.09, *p* = 0.07, − 1.12 ± 0.22, *p* < 0.001).

Pain-related changes in connectivity analysis of the right hypothalamus also revealed significantly reduced pain-related connectivity within the brainstem although in a more restricted pattern. Whilst there were no significant differences between control and interictal migraine groups, significantly reduced hypothalamus pain-related changes in connectivity occurred during the period immediately prior to a migraine in the contralateral midbrain periaqueductal gray matter (PAG), the dlPons bilaterally and in the RVM (Fig. 5B, Table 3). Again, extraction of the magnitude of pain-related changes in connectivity strength changes also revealed that these changes were restricted to the period immediately prior to migraine and did not change during the interictal phase relative to controls (mean ± SEM PPI in controls, interictals, immediately prior to migraine: contralateral PAG 0.51 ± 0.24, − 0.28 ± 0.31, *p* = 0.05, − 1.81 ± 0.80, *p* = 0.001; contralateral dlPons 0.08 ± 0.10, − 0.25 ± 0.16, *p* = 0.07, − 1.46 ± 0.61, *p* < 0.001; ipsilateral dlPons 0.05 ± 0.09, 0.05 ± 0.15, *p* = 0.99, − 1.03 ± 0.20, *p* < 0.001; RVM -0.09 ± 0.16, 0.21 ± 0.22, *p* = 0.27, − 1.73 ± 0.62, *p* = 0.001).

## Discussion

The results of this study demonstrate that in migraineurs, immediately prior to a migraine event, acute orofacial noxious stimuli evoke greater signal changes in cortical and subcortical regions compared with controls, even though the perceived pain intensities are not different. One of these regions, the dlPFC, also displayed decreased whole scan functional connectivity with the hypothalamus and both the dlPFC and hypothalamus displayed reduced pain-related changes in connectivity with brainstem pain modulatory regions. Importantly, these connectivity strength decreases in migraineurs were restricted to the period immediately prior to a migraine attack. These results indicate that immediately prior to a migraine, brainstem pain modulating circuitry control is modulated by the cortex, potentially influencing the on-going activity and/or sensitivity of the brainstem region receiving orofacial afferent drive.

Immediately prior to a migraine, migraineurs demonstrated significantly greater acute pain evoked signal intensity changes compared with controls in four regions, the ipsilateral NAc, contralateral vlPFC and two clusters in the contralateral dlPFC. Interestingly, these differences occurred even though on average, perceived pain intensities were similar in controls and migraineurs throughout the migraine cycle. Pain induced activations of the NAc [34, 35], vlPFC [36] and the dlPFC [37] have been demonstrated in previous studies. The NAc is associated with the reward system and survival behaviors that reduce the possibility of injury or damage signaled by pain are negatively reinforced [38]. In experimental animal studies, analgesic responses can be evoked by injections of morphine into either the PAG or NAc [39, 40] and we have previously shown in humans that the NAc is involved in conditional pain modulation (CPM) analgesia [41]. Although the NAc receives input from the PFC and projects indirectly to the PAG [42], we found no differences in either whole scan or pain-related changes in connectivity between the NAc and other brain regions. Similarly, the vlPFC also displayed significantly greater activation during acute noxious stimuli in migraineurs but no difference in whole scan or pain-related changes in connectivity. Pain that is controllable evokes greater vlPFC activation compared to pain that is not controllable and vlPFC activation occurs when individuals are instructed to use a reappraisal strategy to emotionally disengage from a threatening stimulus [43, 44]. These reports raise the prospect that in our study, migraineurs may be processing the perceived control over the acute pain experience or another aspect of pain other than being involved in descending modulatory control.

In striking contrast to the NAc and vlPFC, the dlPFC displayed significant differences in signal intensity and both whole scan and pain-related changes in connectivity, specifically during the phase immediately prior to a migraine attack. While the dlPFC is typically known for its role in several brain networks such as cognitive processes and working memory [45–47], it has also been established as a key region involved in pain processing and pain modulation [30, 48]. It has been proposed that this region may exert active control on pain perception through modulation of corticosubcortical and corticocortical pathways [30]. Previous fMRI migraine studies have demonstrated increased activation of the dlPFC during pain [37] and we have previously shown that dlPFC activation and connectivity strength with the brainstem is associated with CPM analgesia [41]. In addition, a recent study reported decreased resting state functional connectivity between the dlPFC and PAG in migraineurs, although this study only investigated the interictal phase of migraine [49]. Furthermore, whilst this investigation did not explore the molecular mechanisms underpinning pain processing in migraine, the effects reported here may involve the release of endogenous opioids. This would be consistent with experimental animal and human studies which have demonstrated that endogenous analgesia is often mediated by the descending serotonergic pathways [50] and likely contains an opioidergic action [51]. Endogenous top-down controls projecting to the spinal cord have a role in controlling spinal processing of incoming nociceptive inputs and consequently the intensity of pain in humans [52].

We found that increased activation of the dlPFC in migraineurs immediately prior to a migraine was associated with reduced whole scan connectivity with other pain processing regions such as the VP thalamus, orbitofrontal cortex and also the hypothalamus. The connectivity changes with the hypothalamus were of particular interest since our original hypothesis was that the hypothalamus would be involved in modulating the overall sensitivity of the brainstem. Whilst we did not find differences in hypothalamic signal intensity changes during noxious stimuli in migraineurs, the reduced dlPFC-hypothalamus whole scan connectivity suggests altered function of this pathway in migraineurs. The decrease in hypothalamic connectivity was located in the same lateral hypothalamic region in which we have previously shown significantly reduced on-going blood flow in migraineurs, specifically during the period immediately prior to a migraine attack [17]. Experimental animal tract tracing investigations have shown that the PAG, in particular the ventrolateral PAG column, receives projections from the lateral hypothalamus [53] and activation of this hypothalamic region can produce analgesia, likely mediated by the PAG [54].

The hypothalamus has been implicated as a critical region in migraine initiation and maintenance through its strong cortical connections and exertion over subcortical regions involved in descending pain modulation [9, 55]. Consistent with this idea, we found reduced dlPFC-hypothalamus whole scan connectivity and reduced pain-related changes in connectivity between the lateral hypothalamus and the PAG, dlPons and RVM immediately prior to migraine. Interestingly, whilst we did not find altered whole scan connectivity between the lateral hypothalamus and these brainstem sites, in our previous investigation we reported significantly reduced resting state connectivity between the lateral hypothalamus and these brainstem sites [17]. This difference is likely due to the fact that the “whole scan” connectivity reported in this study was derived from a scan during a series of noxious stimuli and subjects were aware prior to the scan that noxious stimuli were to be administered. It may be that knowing that noxious stimuli are about to be administered, significantly changes hypothalamus-brainstem integration in migraineurs only in the period immediately prior to a migraine attack.

It is well-established that the PAG modulates incoming noxious inputs at the SpV via a projection with the RVM [9, 56]. Within the RVM, distinct populations of neurons termed “off” and “on” cells can inhibit or facilitate neurotransmission at the SpV [7, 57] and in pain-free controls the balance between these cells regulate nociceptive thresholds [58]. In individuals with chronic pain, it has been suggested that there is a shift in pain-modulation system functioning, such that it favors pro-nociception [59]. Indeed the persistence of pain may be attributable to mechanisms including a serotonergic modulatory system in which the RVM is involved in the maintenance as opposed to the establishment of chronic pain [60]. An important RVM mediated serotonergic role for bidirectional pain modulation has been established, and pro-nociception is likely facilitated by excitatory action of serotonin receptors at the spinal cord [59, 61]. It is possible that in migraineurs, as a migraine approaches the balance of this PAG-RVM-SpV system moves towards one that favors pro-nociception and when an acute noxious stimulus is delivered, the connectivity within this brainstem circuitry is subsequently altered. This is consistent with our previous report of reduced acute-pain connectivity between the RVM and SpV in migraineurs immediately prior to a migraine [5].

Interestingly, whilst the lateral hypothalamus displayed significant pain-related changes in connectivity with the PAG and RVM, the dlPFC displayed significant changes with the SRD, RVM and SpV, but not the PAG. This suggests that in addition to altered hypothalamic inputs to PAG-RVM-SpV circuitry, SpV function may also be modulated by projections from the dlPFC either directly or via the RVM or the SRD. Experimental animal studies have shown that the SRD is critical for CPM analgesia expression [62] and we have shown in humans that CPM responsiveness is associated with altered activity in the SRD as well as the dlPons [63]. We have also shown that reduced resting dlPFC-SRD connectivity strength is associated with greater CPM analgesia [41]. It remains unknown if there is a direct neural connection between the SRD and dlPFC in humans and although one rodent tract-tracing investigation did not find a prefrontal-SRD projection [64], another study did [65]. We also found altered decreases in pain-related changes in dlPFC-dlPons and hypothalamus-dlPons connectivity in migraineurs immediately prior to a migraine. The dlPons, more specifically the region of the parabrachial nucleus, is a major target of lamina 1 neurons in the dorsal horn, including those receiving inputs from the orofacial region [66, 67] and it has been shown that inhibiting the parabrachial region results in altered on-going activity in the RVM [68].

While we are confident in the robustness of our findings, there are several limitations that require consideration. Firstly, alterations in dlPFC function may reflect general processing of noxious stimuli given its role in multiple pain related processes and not the modulation of incoming noxious information as we have proposed. Using connectivity measures we cannot assess the direction of information flow, however, given the strong changes in dlPFC connectivity with brainstem regions with well-established roles in pain modulation, we suggest our interpretation of the results are the most plausible. Secondly, the relatively low spatial resolution of the fMRI images presents difficulties in accurately localizing each cluster to a specific nucleus or region within the brainstem and cortices. We used whole brain and brainstem atlases to identify and define the location of each significant cluster and our clusters overlap with regions demonstrated to be involved in nociceptive transmission within the literature and particularly the descending pain modulatory pathway. Thirdly, given the difficulties involved with capturing the 24-h phase immediately prior to a migraine, the sample size collected for this phase is smaller than the interictal phase. The use of uncorrected thresholds for the brainstem connectivity analyses also raises the prospect of Type II errors although we used cluster-based correction and a minimum contiguous cluster extent to limit this as a potential issue. Increasing this sample size of the phase immediately prior to a migraine in future studies to validate our findings and improve study power would be highly desirable, although difficult. Furthermore, we did not have a sufficient sample size for the purpose of performing analyses over the entire migraine cycle at an individual subject level. Finally, analgesic medications have been demonstrated to affect pain modulation in the brain [69], however, only 28% of migraineurs were taking analgesic medication during a migraine. Despite this, we are confident analgesic medication use did not play a significant role in our study, since we found no differences when comparing migraineurs that did and did not take any analgesics.

## Conclusions

Overall, our data reveals that immediately prior to a migraine, the dlPFC and hypothalamus exhibit altered descending influence, as evidenced by reduced connectivity strengths, across brainstem structures involved in processing and modulating incoming noxious inputs. These brainstem structures include those in the classic PAG-RVM-SpV analgesic circuit as well as the SRD-SpV loop responsible for CPM analgesia. Curiously, the occurrence of these changes was independent of overall perceived pain intensity and applied stimuli temperature. Our findings support the theory that increased activation of cortical brain regions is reflective of altered SpV modulation by descending circuits which may enable increased on-going neural traffic or external triggers to initiate a migraine and evoke head pain [4]. The findings support the idea that central changes in pain circuits may be involved in the generation of a migraine attack. A greater understanding of how these functional alterations of the descending pain modulation pathway contribute to migraine initiation and maintenance may lead to the development of effective tailored therapeutic strategies, that may target higher cortical areas to alleviate pain in a timely manner before the onset of a migraine attack.

## Data Availability

The datasets used and analyzed during the current study are available from the corresponding author on reasonable request.

## Abbreviations

MRI: magnetic resonance imaging
fMRI: functional magnetic resonance imaging
PPI: psychophysiological interaction analysis
NAc: nucleus accumbens
vlPFC: ventrolateral prefrontal cortex
dlPFC: dorsolateral prefrontal cortex
